# Aging impacts isolated lymphoid follicle development and function

**DOI:** 10.1186/1742-4933-8-1

**Published:** 2011-01-07

**Authors:** Keely G McDonald, Matthew R Leach, Conway Huang, Caihong Wang, Rodney D Newberry

**Affiliations:** 1 Department of Internal Medicine, Washington University School of Medicine, St. Louis, Missouri 63110, USA; 2 University of Texas Southwestern Medical School, Austin Texas, 78701, USA

## Abstract

**Background:**

Immunosenescence is the age-related decline and dysfunction of protective immunity leading to a marked increase in the risk of infections, autoimmune disease, and cancer. The majority of studies have focused on immunosenescence in the systemic immune system; information concerning the effect of aging on intestinal immunity is limited. Isolated lymphoid follicles (ILFs) are newly appreciated dynamic intestinal lymphoid structures that arise from nascent lymphoid tissues, or cryptopatches (CP), in response to local inflammatory stimuli. ILFs promote "homeostatic" responses including the production of antigen-specific IgA, thus playing a key role in mucosal immune protection. ILF dysfunction with aging could contribute to immunosenescence of the mucosal system, and accordingly we examined phenotypic and functional aspects of ILFs from young (2 month old) and aged (2 year old) mice.

**Results:**

We observed that aged mice have increased numbers of ILFs and increased numbers of structures corresponding to an early stage of CPs transforming into ILFs. The cellular composition of ILFs in aged mice is altered with a smaller B-lymphocyte population and an increased T-lymphocyte population. The ILF T-lymphocyte population is notable by the presence of CD4+ CD8αα+ T-lymphocytes, which are absent from the systemic compartment. The smaller B-lymphocyte population in ILFs from aged mice is directly correlated with decreased mRNA and protein expression of CCL20 and CXCL13, two chemokines that play crucial roles in recruiting B-lymphocytes into ILFs. Aged mice had elevated levels of serum and fecal immunoglobulins and despite the decreased B-lymphocyte population, ILFs from aged mice displayed increased IgA production. The immunoglobulin repertoire was skewed in aged mice, and ILFs demonstrated a repertoire usage similar to that of the systemic pool in both young and aged mice.

**Conclusions:**

Here we observed that ILF development, cellular composition, and immunoglobulin production are altered with aging suggesting that ILF dysfunction contributes to mucosal immunosenescence.

## Background

Immunosenescence is the age-related decline and dysfunction in protective immunity with serious clinical consequences [1-4]. With aging, bacterial and viral infections in the lungs, skin, and urinary tract become more common [5-7]. Compounding this susceptibility to infection, the rates of seroconversion after prophylactic vaccination decrease proportionally with advancing age [8,9]. Related to the decreased ability to mount effective immune responses to pathogens, immunosenescence also leads to a decline in effective immune surveillance potentiating an increased incidence of malignancy [10]. Finally, immunosenescence is not only associated with declining host immune competence, but also with immune dysregulation manifested by an increased incidence in autoimmune and chronic inflammatory disorders with increasing age [11].

Despite the earlier thoughts that the mucosal immune compartment was largely unaffected by aging, the mucosal immune response is now believed to be compromised in old animals and elderly humans [12-14]. The impact of mucosal immunosenescence is highlighted by epidemiological studies demonstrating a marked increase in mortality due to gastrointestinal infections in the elderly in comparison to young adults [13]. Likewise, age is also an important risk factor for colon cancer, the third most deadly cancer in the United States. Coincident with this decline is an increased incidence of individuals diagnosed with inflammatory bowel disease in their seventh decade of life, thus demonstrating a tendency toward the development of inappropriate mucosal immune responses with aging [15,16].

The mucosal immune system is a complex network generating immune responses that both protect the host and mitigate potential damage due to uncontrolled inflammation [17,18]. In the gastrointestinal tract this system includes diffuse effector sites, such as the intestinal lamina propria (LP) and the intraepithelial lymphocyte (IEL) compartment, as well as organized lymphoid structures that are collectively referred to as the gastrointestinal-associated lymphoid tissue (GALT). Isolated lymphoid follicles have recently become appreciated as distinct members of the GALT. ILFs resemble Peyer's patches (PPs), the most widely studied lymphoid structure in the small intestine, in architecture and cellular composition. Like PPs, ILFs can possess germinal centers and an overlying follicle-associated epithelium (FAE) containing M cells [19]. In contrast to PP, whose formation is developmentally driven, with early vital events occurring only during embryogenesis, ILFs develop after birth and arise from nascent lymphoid tissues, or cryptopatches (CPs), in response to luminal stimuli including alterations in the intestinal microbiota [19,20]. The adult murine intestine contains ~1000 CP, and thus in comparison to the relatively small (~10) fixed number of PP, there is potentially a much greater number of the ILFs that can contribute to mucosal immune responses. The function of CP and ILFs are incompletely understood. ILFs, but not CP, are known to act as sites for the induction of adaptive immune responses [21], and studies of young animals demonstrate that ILFs function in a compensatory manner, promoting 'homeostatic' responses to local inflammatory stimuli including the production of antigen specific IgA [22]. Therefore a dysfunction in ILF development or function with aging has the potential to contribute to the immunosenescence of the intestinal immune system in a substantial way.

To gain insight into the role the ILFs play in intestinal immunosenescence, we examined the phenotypic and functional aspects of ILFs from aged and young mice. To our surprise, we observed an increase in the number of structures corresponding to transitioning CP and ILFs with aging. However, consistent with immune dysfunction with aging, we found that ILFs from aged mice had a smaller B-lymphocyte population and an increased T-lymphocyte population when compared with their young counterparts. This finding correlated with decreased expression of the chemokines CXCL13 and CCL20, known to be important in recruiting B-lymphocytes into ILFs, and decreased expression of their corresponding ligands, CXCR5 and CCR6, in ILFs of aged mice. Further analysis of the ILF T-lymphocyte population in aged mice revealed additional abnormalities characterized by the presence of a unique CD4^+ ^CD8αα^+ ^T-lymphocyte population. In agreement with prior observations, we found that the serum immunoglobulin levels in the aged mice were significantly increased and that fecal IgA levels were elevated with aging. Consistent with ILFs contributing to dysfunction of the mucosal immune system with aging, we observed that ILFs from aged animals had elevated production of IgA. Evaluation of the B-lymphocyte immunoglobulin repertoire revealed that the ILF B-lymphocyte population was reflective of the systemic B-cell population and developed a skewed V_H _usage in aged mice. These observations indicate that ILFs are altered with aging and that ILF dysfunction contributes to mucosal immunosenescence.

## Results

### ILF formation is augmented at an early stage with aging

ILFs and CP are components of a spectrum of lymphoid aggregates that have been termed solitary intestinal lymphoid tissue (SILT) [20]. CPs are clusters of unique lymphoid tissue inducer like cells lacking expression of surface markers defining mature bone marrow derived lineages. These clusters act as nascent lymphoid tissues giving rise to ILFs in response to inflammatory stimuli. As CP progress to become ILFs they become infiltrated with a substantial population of dendritic cells [23], and accordingly the presence of CD11c+ clusters can be used to identify stages of SILT ranging from CP progressing to immature ILFs to fully developed mature ILFs. In comparison to CP, ILFs are larger, more complex cellular aggregates containing mature B- and T-lymphocytes. ILFs can range from lymphocyte containing cellular clusters encompassing the crypt and extending up the villi to more organized aggregates having a follicular appearance possessing an overlying follicle associated epithelium (FAE). ILFs in all stages of development can be identified as clusters of B220+ cells and the more developed or mature ILFs can be identified by their nodular appearance and FAE identified with *Ulex europaeus *(UEA-I) staining.

**Figure 1:**
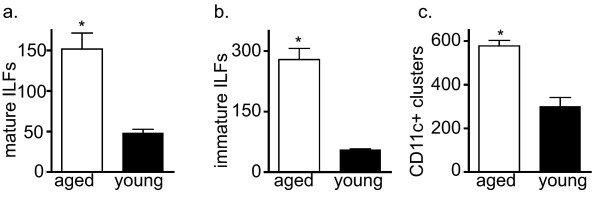
**ILF formation is augmented at an early stage with aging**. To evaluate if the process of ILF development was altered with aging, whole mount techniques were used to identify well developed SILT containing a follicle associated epithelium indicative of mature ILFs (panel a), poorly developed or immature ILFs comprising a loose cluster of B-lymphocytes (panel b), and nascent lymphoid tissues transitioning into ILFs as identified by CD11c+ clusters (panel c) in young and aged mice. Aged mice had significantly increased numbers of mature ILFs (panel a), immature ILFs (panel b), and CD11c+ clusters (panel c), indicating that all stages of CP transitioning into ILFs are augmented with aging. n = 3 or more mice in each group. * = p < 0.05.

To begin assessing the impact of aging on ILF formation, we examined the number of SILT at various stages of development in young and aged mice. We noted that the numbers of mature ILFs (mILFs), or fully developed ILFs, in the intestines of the aged mice were significantly higher than those of the young mice (Figure 1a). Consistent with the increased number of mILFs, we observed that the aged mice had a significantly increased number of immature ILFs (iILFs), or B-lymphocyte containing structures that are not fully developed, when compared to young mice (Figure 1b). We also observed an increased number of CD11c+ clusters identifying components of the SILT spectrum encompassing CP transitioning to iILFs and mILFs (Figure 1c). Prior reports indicate that the numbers of CP are relatively static, and we observed no difference in the numbers of CP between aged and young animals (not shown). Studies in young animals indicate that the influx of CD11c+ cells in response to inflammatory stimuli is an early event in the transformation of CPs into ILFs [23]. In conjunction with our findings of augmented ILF development with aging, this suggests that during aging the intestine is subject to chronic inflammation.

### The cellular composition of the ILFs is altered with aging

**Figure 2:**
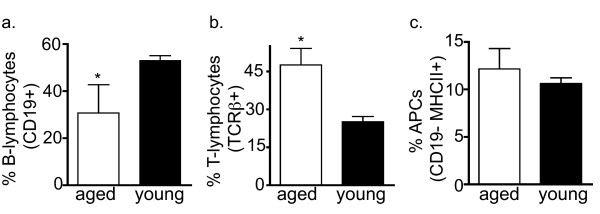
**ILFs from aged mice have an aberrant cellular composition**. The cellular composition of ILFs isolated from aged and young mice was evaluated using multi-color flow cytometry. ILF cellular populations from aged mice had a significantly decreased population of B-lymphocytes (panel a) and a significantly increased population of T-lymphocytes (panel b). The population of antigen presenting cells (APCs) was not significantly different between aged and young mice. n = 3 or more mice in each group. * = p < 0.05.

**Figure 3:**
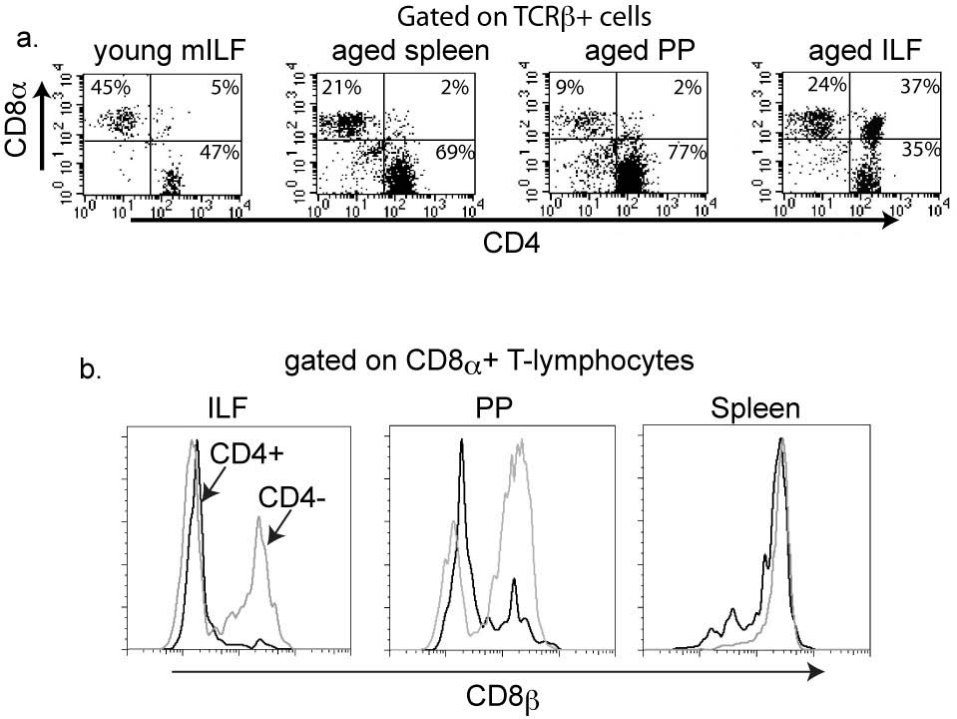
**ILFs from aged mice contain a population of CD4+ CD8αα+ T-lymphocytes**. ILF, PP, and splenocyte populations from young and aged mice were isolated and evaluated by multi-color flow cytometry for the presence of CD4+ and CD8α+ T-lymphocytes (panel a). ILFs from aged mice contained a significant population of CD4+ CD8α+ T-cells, which was largely absent from the spleen and PP of aged mice and from the ILFs of young mice (panel a). Further analysis of the CD4+ CD8α+ population within ILFs from aged mice revealed that this population was CD8β-, and therefore CD8αα+ (black line histogram panel b). This CD4+ CD8αα+ T-lymphocyte population was also present in PP from aged mice, but absent from the splenocyte population of aged mice (black line histogram panel b). Within the ILF and PP population from aged mice the CD4- CD8α+ T-lymphocytes were both CD8β+ and CDβ-, while in the spleen this population was only CD8β+ (gray line histograms panel b). Data shown are representative of two or more experiments.

The cellular composition of ILFs in young mice resembles that of PP and lymph nodes, and includes B-lymphocytes, T-lymphocytes (CD4 or CD8 single positive), and dendritic cells [19-21]. To gain insight into the function of ILFs in aging, we examined the cellular compositions of ILFs in aged and young mice using flow cytometry. We found that in comparison to young mice, the aged mice had a smaller population of B-lymphocytes (CD19+ cells) and an increased population of T-lymphocytes (TCRβ+ cells) (Figure 2a, 2b). We observed no significant differences in the antigen presenting cell population (CD19-, MHC II+) from the young and aged mice (Figure 2c). Further analysis of the increased T-lymphocyte population from aged mice revealed the presence of a unique CD4^+ ^CD8α+ (double positive) TCRβ+ population (Figure 3a). These double positive T lymphocytes were rare in spleen and the PPs of the aged mice. The double positive cells represented 2.33 +/- 0.145% and 19.05 +/- 3.55% (mean +/- SD) of the total ILF cellular population falling within the lymphocyte gate for the young and aged mice, respectively. CD8 is a transmembrane glycoprotein that binds to class I MHC proteins and serves as a co-receptor for TCR. It forms a dimer, most commonly consisting of CD8α and CD8β (CD8αβ+ heterodimer). Less commonly, homodimers of two CD8α chains are also expressed. Further analysis of the CD4+CD8α+ cells from ILFs of the aged mice revealed that these cells did not express CD8β, and hence are CD4+ CD8αα+ TCRβ+ T-cells (Figure 3b). Likewise the minor population of CD4+ CD8α+ T-cells from the PP of aged mice were CD8β-, while the spleen of aged mice did not contain a CD8α+ CD8β- population (Figure 3b). The ILFs and PP of aged mice also contained CD4- CD8αα+ and CD4- CD8αβ+ populations, while CD4- CD8αα+ populations were largely absent from the spleen (Figure 3b).

### ILFs from aged mice have altered expression of chemokines and chemokine receptors required for ILF development

**Figure 4:**
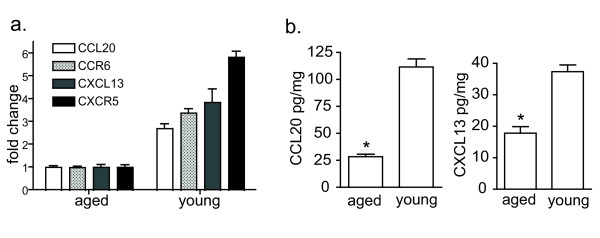
**ILFs from aged mice express lower levels of chemokines and chemokine receptors demonstrated to play a role recruiting B-lymphocytes into ILFs**. RNA was isolated from pooled ILFs from aged and young mice. The mRNA expression of CCL20, CXCL13, CCR6, and CXCR5 was evaluated using real time PCR (panel a), and the protein expression for intestinal CCL20 and CXCL13 was measured by ELISA (panel b). ILFs from aged mice showed decreased mRNA expression of chemokines CCL20 (~3 fold), CXCL13 (~4 fold) and their respective receptors CCR6 (~3.5 fold), CXCR5 (~6 fold) (panel a), and significantly decreased intestinal protein expression of CCL20 and CXCL13 (panel b). * = p < 0.05.

We previously reported that CC chemokine receptor-6 (CCR6) and its ligand CCL20 are highly expressed within ILFs and that B-lymphocytes are the largest CCR6-expressing population within ILFs [18]. Furthermore, CCR6-sufficient B-lymphocytes are essential for the formation of ILFs [18]. Prior studies have demonstrated that CXC chemokine receptor-5 (CXCR5) and its ligand, CXCL13 or B-lymphocyte chemoattractant (BLC), are required for the migration of B lymphocytes to organized lymphoid follicles and the development of PP and SILT [23-25]. To assess the roles of these chemokines and their receptors in the age-related augmentation of SILT formation and aberrant ILF cellular populations, we examined the mRNA expression of CCR6, CCL20, CXCR5, and CXCL13 in ILFs using real-time polymerase chain reaction. We noted that the mRNA expression of chemokines CCL20, CXCL13, and their receptors CCR6 and CXCR5 were significantly lower in ILFs of the aged mice than in those of the young mice, which correlated with decreased expression of CCL20 and CXCL13 protein in the intestine of aged mice (Figure 4). These factors are essential for B-lymphocyte homing to lymphoid follicles, and their decreased expression is in agreement with the smaller B-lymphocyte population in the ILFs of aged mice.

### Aged mice produce higher levels of systemic and mucosal immunoglobulins

The literature on immunoglobulin production in aged animals and humans is limited and somewhat conflicting. Some studies have reported increases or no change in the serum IgA levels in old animals and humans [26-29]. A few studies also reported the absence of age-related differences in the amounts of nonspecific immunoglobulins secreted into the intestinal lumen *in vivo *or into the medium by cultured duodenal biopsies [28-31], while other studies observed that the production of antigen-specific IgA is diminished with aging [14,32,33].

**Figure 5:**
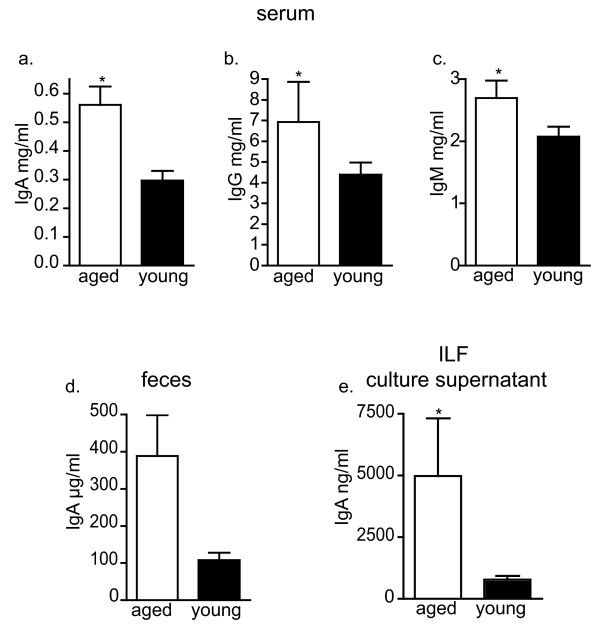
**Aged mice have elevated serum and mucosal immunoglobulin levels and elevated IgA production by ILFs**. Serum and feces were collected from aged and young mice and immunoglobulin levels were measure by ELISA. Aged mice had significantly elevated serum IgA (panel a), IgG (panel b) and IgM (panel c). Aged mice also displayed elevated fecal IgA (panel d) and culture of ILF cellular populations revealed that IgA production was increased in ILFs from aged mice (panel e). n = 12 or more for serum Ig measurements, n = 3 or more for fecal Ig levels. n = 2 or more independent experiments for culture supernatant Ig levels. * = p < 0.05

We observed significantly elevated levels of IgA, IgG and IgM in the serum of the aged mice (Figure 5a-c), and increased levels of IgA in the feces of aged mice (Figure 5d). ILFs preferentially induce B-lymphocytes to become IgA producing plasma cells [22]. To evaluate the contribution of the ILFs to the increase in fecal IgA with aging, we isolated and cultured ILF cellular populations and evaluated the IgA levels in the culture supernatant. Consistent with ILFs contributing to the increase in fecal IgA with aging, we found that ILFs from aged mice had elevated IgA production (Figure 5e).

### ILF immunoglobulin repertoire reflects the systemic compartment and becomes skewed with aging

**Figure 6:**
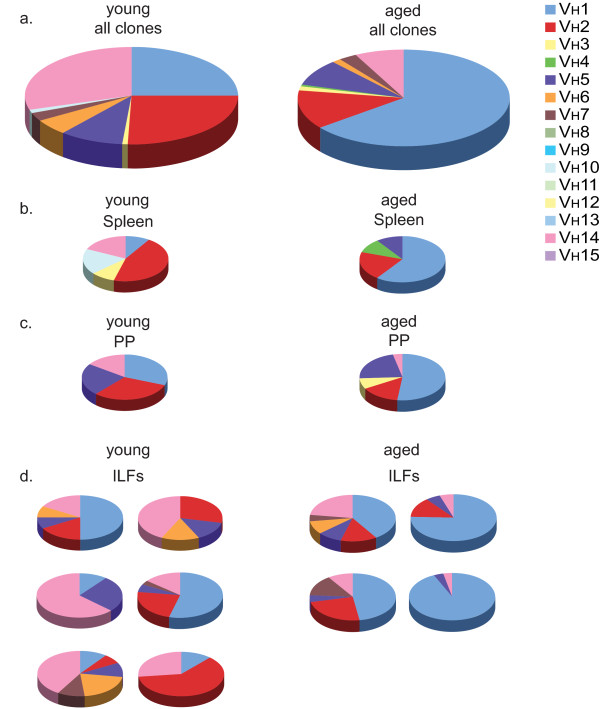
**ILFs from young and aged mice contain a B-lymphocyte repertoire that is reflective of the systemic B lymphocyte pool; the B-lymphocyte repertoire becomes skewed with aging**. RNA isolated from the spleen, PP, and individual ILFs from a young and aged mouse was transcribed into cDNA and immunoglobulin genes amplified using a universal V_H _primer and a constant IgM region primer. Gel-purified products were ligated into vectors and individual clones containing the appropriate size insert were sequenced and assigned to a V_H _family. Analysis of all clones identified from the young (left) and aged mice (right) revealed a similar preferential usage of V_H _families with V_H_1, V_H_2, and V_H_14 representing greater than 80% of the VH families used by either mouse (panel a). With aging, the Ig repertoire became more skewed with dominant usage of the V_H_1 family (panel a right). Comparison of the V_H _usage within the spleen (panel b), reflective of the systemic pool, to the PP (panel c) and individual ILFs (panel d) revealed similar V_H _usage between these tissues within aged and young mice and revealed that the Ig repertoire was skewed toward dominant V_H_1 usage in all tissues in the aged mouse (panels b-d right).

The development of ILFs is not completely understood, with some studies suggesting that ILFs develop from the recruitment of peripheral B-lymphocytes and others suggesting that B-lymphocytes within ILFs expand *in situ *to generate follicles. In support of the former possibility we previously reported that individual ILFs from young mice contain a population of polyclonal B-2 B lymphocytes that was reflective of the systemic pool of B-lymphocytes [22]. Consistent with the second possibility, others reported that the predominant immunoglobulin V_H _gene used within individual ILFs in aged activation-induced cytidine deaminase (AID)-deficient mice varied, suggesting that different B-lymphocytes expand within individual ILFs skewing the immunoglobulin repertoire usage such that it was not reflective of the systemic compartment [34]. While these two possibilities are not mutually exclusive, they could suggest different pathways by which the number of ILFs would expand during aging; with the former implying that the increased number of ILFs occurs by recruitment of B-lymphocytes representative of the systemic pool and the latter that increased ILF development results from expansion of B-lymphocytes within the ILFs, possibly reflective of a B-lymphocyte intrinsic process. To address these possibilities, we analyzed the immunoglobulin heavy chain repertoire usage within the spleen, PP, and individual ILFs from young and aged mice. We observed that the V_H _usage within ILFs from young mice was diverse, indicative of a polyclonal population, paralleling that of the spleen, which is representative of the systemic pool (Figure 6a-d). Likewise, in the aged mouse the V_H _usage within all the ILFs examined was highly reflective of that seen in the spleen, and thus reflective of V_H _usage within the systemic compartment (Figure 6a-d). While both young and old mice had a similar usage of V_H _families, with V_H_1, V_H_2, and V_H_14 representing greater than 80% of the V_H _families used by both groups, the usage of an individual V_H _family became more skewed with aging as the V_H_1 family became the predominant family used in each tissue, representing over 60% of the V_H _family usage overall.

## Discussion

Immunosenescence is a state of dysregulated immune function contributing to the increased susceptibility of the elderly to infection, autoimmune diseases, chronic inflammatory diseases, and cancer [1-4]. The majority of studies on immunosenescence have examined the systemic immune system and support that systemic immunity is affected with aging. Studies of the impact of aging on the mucosal immune system are less numerous, however emerging data more directly suggests an affect of aging on the mucosal immune system. Age greater than 65 has been identified as an independent risk factor for the development of *C. difficle *colitis [35], an enteric infection whose recurrence and clinical course are related to the production of anti-toxin specific IgA in the intestinal lumen [36]. Aging is associated with a decreased ability to produce antigen specific immunoglobulins, including IgA, following mucosal immunization [32,37-39], implying a direct impact of aging on the mucosal immune system, with a resultant increased risk of enteric infection. While it is becoming more clear that the mucosal immune system is affected by aging, the components of the mucosal immune system that are affected by aging and the functional consequences of these changes are still largely unexplored.

Organized lymphoid tissues play a crucial role in immunity by providing sites for the efficient interactions of antigens, antigen presenting cells, and lymphocytes. These structures are essential for the initiation of primary immune responses, and the regulated environment they provide is believed to be necessary to prevent inappropriate immune responses. Traditionally the organized lymphoid tissues of the intestine were thought to be limited to secondary lymphoid structures, or Peyer's patches (PP). The foundations for PP formation only occur during embryogenesis, and therefore the number and positioning of PP are fixed throughout life [40-43]. Recent studies have shown that the adult intestine contains an additional population of organized lymphoid tissues, or solitary intestinal lymphoid tissues (SILT). SILT are a continuum of lymphoid tissues that have a unique plasticity allowing for the development and regression of a fully functional lymphoid tissue that can mediate homeostatic responses [17,19-21]. Within this continuum, CP act as nascent lymphoid tissues that sequentially become invested with dendritic cells and B-lymphocytes, to become ILFs [23]. Poorly developed, or immature ILFs (iILFs), can subsequently expand and develop a dome like structure with a follicle associated epithelium (FAE) to become mature ILFs (mILFs), which have the potential to initiate controlled adaptive immune responses to luminal antigens [21]. The murine intestine contains ~1000 SILT, and therefore in contrast to PP which are fixed and more limited in number (~10), SILT have a greater potential to act as sites for the induction of intestinal immune responses and conversely to be affected by immunosenescence.

There is precedence for newly developed lymphoid tissues to contribute to chronic inflammatory and autoimmune pathologies. Inducible lymphoid tissues, are markedly increased in conditions with inappropriate immune responses, such as those seen in inflammatory bowel disease (IBD) [20]. These aberrantly formed or tertiary lymphoid tissues have been proposed to initiate or propagate the inappropriate responses seen in autoimmune and chronic inflammatory conditions and harbor the earliest manifestations of IBD [44,45]. Thus it is possible that ILFs formed aberrantly as a result of immunosenescence might subsequently contribute to immune dysfunction and participate in the increased incidence of IBD seen in the seventh decade of life.

Here we demonstrate that ILF formation is augmented with aging. We observed that the numbers of CPs containing DCs, iILFs, and mILFs were significantly increased in the aged mice. Aged mice were on average 25% larger than their gender and strain matched counterparts; average of 28.4 gm vs. 22.6 gm for 17 month old vs. 11 week old female Balb/c mice (information from the National Institute on Aging rodent colony). While this increase in body size could translate to a proportional increase in the number of ILFs, alone it is not sufficient to explain the three-fold increase in ILFs we observed. Furthermore, the cellular compositions of ILFs from the aged mouse are abnormal with a decreased B-lymphocyte population and the appearance of CD4+CD8+ double positive T-lymphocytes. To assess the factors contributing to the decreased B lymphocyte population in ILFs with aging, we examined the expression of the chemokines CCL20 and CXCL13, which have been demonstrated to play a role in recruiting B-lymphocytes into SILT [18,23,25]. Similar to the process of ILF formation, the production of CCL20 is induced by inflammatory conditions, and CCR6, the only identified receptor of CCL20, is expressed by B-lymphocytes [46-49]. CCR6 is required for the development of ILFs and CCR6 deficient B-lymphocytes have reduced ability to localize to PPs and ILFs [18]. In a similar fashion, CXCR5 expression by B-lymphocytes is required for the development of ILFs, and ILF dendritic cells were identified as sources for CXCL13 production to support ILF development [23,25]. CXCL13 not only recruits CXCR5+ B-lymphocytes, but also induces the expression of lymphotoxin (LT) by antigen-naïve B-lymphocytes, which further induces the production of CXCL13 by lymphotoxin beta receptor (LTβR) expressing stromal cells [24]. In addition, LT expression by B-lymphocytes is required for the development of SILT with large follicles, or mature ILFs [17]. Since CCR6+ B lymphocytes are also CXCR5+ [50], CCL20 and CXCL13 may act synergistically to recruit antigen naïve B-lymphocytes into ILFs and promote the development of mature follicles via LT/LTβR pathways. We observed a significant decrease in the expression of CCL20, CXCL13, and their respective receptors CCR6 and CXCR5 in the ILFs of aged mice when compared with those of the young mice. The decreased expression of CCL20 and CXCL13 and resultant decreased B-lymphocyte population in the ILFs of the aged mice is consistent with these previously observed functions of CCL20 and CXCL13. The combination of a decreased expression of these chemokines and a decreased ILF B-lymphocyte population with a paradoxical increase in the number of ILFs suggests that alternative pathways may be emphasized in ILF development during aging.

Peripheral T-lymphocytes are generally grouped into two subsets on the basis of the mutually exclusive cell surface expression of the CD4 and CD8 co-receptors. The expression of the CD4 or CD8 co-receptors by mature peripheral T-lymphocytes defines function specific differences (helper vs. cytolytic function respectively) as well as differences in major histocompatibility complex (MHC) restriction (MHC II vs. MHC I respectively). An exception to this generalization in which mature peripheral T-lymphocytes express both CD4 and CD8 co-receptors has been noted across many species [51-54]. The double positive cells can be grouped on the basis of CD8β expression, i.e. CD4+CD8αα+ and CD4+CD8αβ+. There is evidence that the CD4 and CD8 co-receptors are functional in these double positive cells [55-57]. Regulating these double positive cells would be complex, as they could be more prone to generating inappropriate responses due to the multiplicity of receptors. The origin and function of the double positive cells is unclear. They have been suggested to be 1) recent thymic emigrants that are precursors of single positive cells, 2) single positive cells that have upregulated expression of the reciprocal coreceptor upon activation, 3) extrathymic T lymphocytes generated within the intestine, at the sites of CP, in response to the involution of the thymus with aging, or 4) T lymphocytes that were aberrantly selected in the thymus [53,54,56,58,59]. The double positive cell were less common in PP and rare in the spleen of aged animals, supporting the preferential localization of this rare T-lymphocyte population to the intestinal mucosa. Double positive T-lymphocytes have been noted to accumulate in the peripheral blood and intraepithelial lymphocyte compartment with aging [54,55,59,60] and in target organs affected in autoimmune and chronic inflammatory conditions such as atopic dermatitis, systemic sclerosis, and autoimmune thyroid disease, suggesting that these cells may be responsible for the pathologic immune responses seen in these conditions[59]. Coupled with our observations, this suggests that with aging ILFs become sites of dysregulated immune responses potentially contributing to the increased incidence of IBD with aging.

A characteristic feature of the mucosal immune system is its ability to produce and secrete IgA. Some studies reported the absence of age-related differences in the amounts of nonspecific immunoglobulins secreted into the intestinal lumen *in vivo *or into the medium by cultured duodenal biopsies, while others reported that the level of IgA increased in the intestinal juice from aged mice [31,61]. The sources contributing to lumenal IgA are complex and include both B1 and B2 lymphocytes arising from the systemic immune system, mucosal immune system, and the peritoneal cavity. Mature ILFs are sites for the generation of IgA producing plasma cells from B2 B-lymphocyte precursors in response to lumenal antigens [21,22]. Therefore alterations in ILF development and/or function with aging could impact IgA production and mucosal protection. We observed that with aging IgA was significantly elevated in the serum (~ 2 fold) and further elevated in the feces (~ 4 fold), implying a mucosal source that contributes to these elevated fecal IgA levels. While the relative contribution of each of the above mentioned sources to the elevated mucosal IgA levels can not be determined, the increased number of mature ILFs (~3 fold) seen with aging and the increased production of IgA by ILF cellular populations (~ 5 fold) with aging are consistent with ILFs as contributors to the increased lumenal IgA and suggest that they become a more significant contributor to lumenal IgA with increasing age. The increased IgA seen in the aged ILFs occurs despite the decreased population of ILF B-lymphocytes. In concert with the increased number of ILFs, this further suggests the mucosal immune system becomes dysregulated with aging.

B-lymphocytes are a prominent hematopoietic cellular population within ILFs. The sources of B-lymphocytes that contribute to the emergence of the developing SILT are not completely understood, but they may originate from two sources that are not mutually exclusive and include B-lymphocytes that are recruited from the systemic pool or B-lymphocytes that expand *in situ *to generate ILFs. To better understand how ILFs are generated during aging we evaluated the immunoglobulin repertoire usage within ILFs and compared it to the systemic pool as represented by the immunoglobulin repertoire usage within the spleen. We observed that in both young and aged mice the ILF immunoglobulin repertoire was reflective of that seen in the spleen. Like prior studies we also observed that B-lymphocyte repertoire usage becomes less diverse with aging [62]. However, this skewed repertoire usage was consistent throughout the tissues evaluated in the aged mice further supporting that ILF B-lymphocytes are recruited from the systemic pool. Multiple factors could contribute to the skewing of the B-lymphocyte repertoire, including diminished B-lymphocyte precursors and expansion of oligoclonal B-lymphocyte populations [63]. Skewing of the B-lymphocyte repertoire with aging is associated with poor health [62], and the observation that this also occurs within the mucosal immune compartment is further evidence that the mucosal immune system is impacted by aging and adversely affected by immunosenescence.

## Conclusions

ILFs are recently recognized contributors to mucosal immunity acting as a reservoir of inducible sites for the generation of adaptive immunity including T-lymphocyte dependent and independent IgA responses[21,64]. Emerging epidemiologic observations implicate mucosal immunosenescence as a risk factor for enteric infections, autoimmunity, and malignancy. Here we provide evidence to support these epidemiologic observations and demonstrate that like the components of the systemic immune system, aging impacts these recently recognized contributors to mucosal immunity.

## Methods

### Mice

Female BALB/c mice of indicated age were purchased from National Institute on Aging (NIA) and were housed in a specific pathogen free facility and fed routine chow diet. Young mice were 8-12 weeks old, and old mice were 21-23 months old. Mice were housed for at least 2 weeks prior to experiments to allow the mucosal immune system to accommodate to changes in bacterial flora. No differences in the measured parameters were observed with longer periods of housing. Animal procedures and protocols were carried out in accordance with the institutional review board at Washington University School of Medicine.

### Whole mounts of small intestine

Small intestines were removed intact, flushed with cold PBS, and opened along the mesenteric border. Intestines were mounted, lumen facing up and fixed with cold 10% buffered formalin phosphate (Fisher Scientific) for 1 h at 4°C. Intestines were washed three times in cold PBS, incubated in a solution of 20 mM DTT, 150 mM Tris, and 20% ethanol at room temperature for 45 min, washed three times in cold PBS, and incubated in a solution of 1% H_2_O_2 _for 15 min at room temperature to block endogenous peroxidases. Intestines were washed three times in PBS, followed by incubation in PBS containing 1% BSA and 0.3% Triton X-100 for 30 min. Intestines were incubated with HRP-conjugated lectin from *Ulex europaeus *(UEA-I) (Sigma-Aldrich, St. Louis, MO) in PBS containing BSA and Triton X-100 overnight at 4°C to facilitate the identification of PP and mature ILF (mILF). The following day, intestines were washed three times in PBS, incubated in DAB metal peroxide substrate (Pierce, Rockford, IL) for 15 min, rinsed twice in distilled water, and returned to PBS for further analysis. Under low-power microscopy (25-65X) the following criteria were used to determine the presence of mILF: 1) presence of a nodular structure with size equal to or greater than the width of one villus, 2) nodular structure possessing an overlying dome resembling the FAE of PP, and 3) nodular structures occurring singly or in groups of two (three or more nodules of approximately the same size were considered to be PP).

For anti-B220 and anti-CD11c staining of whole mounts to determine the numbers of immature ILFs (iILFs) and CD11c+ clusters, identifying CP transitioning into ILFs and ILFs, intestines were removed intact, flushed with PBS, opened along the mesenteric border, and mounted as above. Intestines were then incubated three times in HBSS (BioWhittaker, Walkersville, MD) containing 5 mM EDTA at 37°C with shaking for 10 minutes to remove epithelial cells. Intestines were then fixed in 10% buffered formalin phosphate and treated with 1% H_2_O_2 _for 15 minutes at room temperature as above. Intestines were incubated in a solution of 50 mM Tris pH 7.2, 150 mM NaCl, 0.6% Triton-X 100, and 0.1% BSA for one hour at 4°C to block non-specific antibody binding and then incubated with rat anti- mouse B220 antibody or biotin conjugated hamster anti-mouse CD11c (both from BDbiosciences, San Diego, CA) diluted in the above solution overnight at 4°C. Intestines were washed three times in the above solution and incubated with a horseradish peroxidase conjugated goat anti-rat IgG antibody or streptavidin conjugate horseradish peroxidase (Jackson ImmunoResearch Laboratories, West Grove, PA) diluted in the above solution at room temperature for one hour. Intestines were washed three times and incubated in DAB metal peroxide substrate as above. Intestine whole mounts were examined under a dissecting microscope at 25-65X. Immature ILFs (iILFs) were counted as clusters of B220+ cells occurring at the base of villi and not containing an overlying dome. Dendritic cell clusters were counted as clusters of CD11c+ cells occurring at the base of villi [17,20].

### Isolation of cellular populations from spleen, PP, and ILFs

Spleens and PP were removed from BALB/c mice and disrupted by mechanical dissociation. Intestines were flushed with cold PBS, opened along the mesenteric border, and mounted with the lumen facing up in cold PBS, as described above. PP were identified, cut out, and disrupted using mechanical disassociation. Using a dissecting microscope and a blunt-end 26-gauge needle and syringe, the contents of multiple mILFs were aspirated and placed in cold PBS. RBC were lysed from each cellular suspension and then used for flow-cytometric analysis as described below. Average yield of viable mononuclear ILFs cells ranged from 3-7 × 10^5 ^cells/intestine [17].

### ELISAs for immunoglobulins, CCL20, and CXCL13

Feces were collected from individual mouse, diluted 1/10 wet weight to volume with PBS, vortexed into a uniform suspension, and centrifuged at 12,000 rpm for 10 min in a tabletop microcentrifuge, and supernatants were harvested. Coagulated mouse blood samples from individual mice were centrifuged and the sera collected. ILF cellular populations were isolated and cultured for five days as previously described[22]. Immunoglobulin levels were measured in fecal supernatants, serum, and culture supernatants by ELISA as previously described[22]. CCL20 and CXCL13 were measured in protein lysates obtained from sections of intestine from aged and young mice using commercially available assays (R and D systems, Minneapolis, MN) per the manufacturers recommendations.

### Flow Cytometric Analysis

Single cell suspensions, obtained as above were used for flow cytometric analysis. Briefly, single cell suspension were resuspended in PBS with 1% BSA (Fisher Scientific) and 1 mg/ml human IgG (Sandoz Pharmaceuticals, East Hanover, NJ) at 2 × 10^7 ^cells/ml. Cells were incubated with antibodies for 30 min on ice in the dark. Cells were washed in PBS and fixed with 1% paraformaldehyde in PBS. Events were analyzed using a FACScan cytometer (BDbiosciences) retrofitted with a second laser. Data acquisition was performed using Cellquest (BDbiosciences) and Rainbow (Cytek, Fremont, CA) software. Dead cells were excluded based on forward and side light scatter. Gates for positive staining were defined such that 1% of the analyzed population stained positive with the appropriate isotype control antibodies (all from BD Biosciences).

### RNA isolation and real-time polymerase chain reaction

Mature ILFs were isolated from both young and aged mice using a dissection microscope and 26-gauge blunt needle as described above. The aspirates containing the overlying FAE, stromal elements, and mononuclear cells from the same mouse were pooled together. RBCs were lysed from each cellular suspension. RNA was isolated using the Arcturus PicoPure RNA isolation kit (Applied Biosystems, Foster City, CA), and treated with DNase I (Ambion, Austin, TX) to remove contaminating DNA. cDNA was synthesized from 2 μg of total RNA using Superscript II RNase H- reverse transcriptase (Invitrogen, Carlsbad, CA). Expression of targets was detected by real time PCR using ABI prism 7700 sequence detection system and SYBR Green PCR master mix (Applied Biosystems). The following primers were used for detection of the targets, forward primers are listed first followed by reverse primers: 18S 5'-CGGCTACCACATCCAAGGAA-3' and 5'-GCTGGAATTACCGCGGCT-3', β-actin 5'-GCTTCTTTGCAGCTCCTTCGT-3' and 5'-ATATCGTCATCCATGGCGAAC-3', CCL20 5'-TGATGCTTTTTTGGGATGGAA-3' and 5'-AGCCTTCAACCCCAGCTGT-3', CCR6 5'-TGTTCTGCTATCTGTTCATTATCAAGA-3' and 5'-CACGACTCGGATGGCTCTGT-3', CXCR5 5'-GGGCCCCTGTCTGTTTCTGT-3' and 5'-GCCCAAGCTCGAGTTGGAT-3', and CXCL13 5'-CAGAATGAGGCTCAGCACAGC-3' and 5'-CAGAATACCGTGGCCTGGAG-3'. Samples were measured in triplicate. Relative quantitation of target expression using 18S or β-actin as a housekeeping gene was determined using the comparative CT method as described in the ABI Prism 7700 sequence detection system user bulletin.

### Immunoglobulin heavy chain repertoire analysis

Single cell populations from spleen, PP, and individual ILFs were isolated as described above and the RNA was isolated using the Arcturus PicoPure RNA isolation kit. cDNA was synthesized using the isolated RNA as template as described previously. The immunoglobulin genes were amplified with PCR using a universal VH primer 5'-AGGTSMARCTGCAGSAGTCWGG-3' in combination with an IgM constant region primer 5'- CCCTGGATGACTTCAGTGTTG - 3'[65]. Amplified products were ligated into the pCR-BluntII-TOPO vector (Intivtrogen Corporation, Carlsbad, CA) and plasmids were isolated from individual clones and sequenced. Sequences were analyzed using The International Immunogenetics Information System (IMGT/V-Quest). Four hundred and forty seven individual clones were sequenced of which 298 clones could be definitively assigned to a V_H _family.

### Statistical analysis

Data analysis using Student's *t *test and one-way ANOVA followed by Tukey's multiple comparisons post-test was performed using GraphPad Prism (GraphPad Software). A value of p < 0.05 was used as a cutoff for statistical significance.

## List of abbreviations

CCL20: Chemokine (C-C motif) ligand 20; CCR6: C-C chemokine receptor 6; CP: cryptopatch; CXCL13: Chemokine (C-X-C motif) ligand 13; CXCR5: C-X-C chemokine receptor 5; IBD: inflammatory bowel disease; ILF: isolated lymphoid follicle; iILF: immature ILF; LT: lymphotoxin; LTβR: lymphotoxin beta receptor; mILF: mature ILF; PP: Peyer's patch.

## Competing interests

The authors declare that they have no competing interests.

## Authors' contributions

KM performed whole mount analysis of intestines, flow cytometry of cellular populations, isolation and in vitro culture of ILF cellular populations, and ELISAs. ML performed experiments related to the analysis of the immunoglobulin repertoire and ELISAs for CCL20 and CXCL13. CH and CW performed ELISAs for immunoglobulins and gene expression analysis. CH helped draft the manuscript. RN conceived of the study, participated in the design and analysis of the experiments, and drafted the manuscript. All authors read and approved the final manuscript.
